# Probiotics as a Preventive Strategy for Recurrent Bacterial Vaginosis: A Narrative Review

**DOI:** 10.7759/cureus.89764

**Published:** 2025-08-10

**Authors:** Jessica Arias Valverde, María Laura Alvarado Fernández, María Luisa Alvarado Mora, María Jesús Arias Alvarado, Paula Vanegas Navarro

**Affiliations:** 1 General Medicine, Universidad de Costa Rica, San José, CRI

**Keywords:** bacterial vaginosis, lactobacilli, prevention, probiotics, recurrence

## Abstract

Bacterial vaginosis (BV) is a highly prevalent condition worldwide, with high recurrence rates despite adherence to antibiotic regimens recommended by clinical guidelines, and it has a negative impact on the lives of many affected women. Consequently, there is a growing need to explore alternative treatments that complement or substitute antibiotics to prevent the recurrence of BV. One such approach that has recently been investigated is the use of probiotics. This article aims to develop a narrative review of the general aspects of BV and the most recent evidence regarding the effectiveness of probiotics in preventing BV recurrence, including the most recently studied strains and routes of administration.

## Introduction and background

Bacterial vaginosis (BV) is a condition that arises from an imbalance in the normal vaginal microbiota, which is predominantly composed of various species of *Lactobacillus*, in favor of a flora dominated by opportunistic pathogenic bacteria, which tends to be anaerobic [1-4]. This condition is highly prevalent worldwide, affecting from 20% to 60% of women, depending on the country. The highest prevalence is reported to be in some areas of Sub-Saharan Africa, with moderate prevalence in South and Southeast Asia, Latin America, the Caribbean and the US. Some hypotheses have been proposed to explain why prevalence varies across areas, such as variations in cultural and economic factors within each region, which in turn imply differences in behaviors and lifestyles. These differences, as described later in the article, influence the appearance of BV. Furthermore, depending on resource availability, diagnostic techniques, statistical case registration, and screening policies may vary. Also, these policies vary depending on the clinical guidelines used [4].

BV is considered to be the most common cause of abnormal vaginal discharge, though it may present with or without symptoms [1-4]. Addressing this pathology is relevant not only because of its negative impact on quality of life but also due to the complications it may entail [1,2].

Current management guidelines primarily recommend oral or vaginal antibiotic therapy. However, a significant proportion of patients experience recurrence after completing the treatment course, and this may include up to 50% of them [5]. Due to this issue, various alternatives have been explored either to replace or to complement antibiotic therapy in an effort to reduce BV recurrence rates. Among these alternatives, the use of different probiotics has gained traction, with promising results reported in literature [6-8].

## Review

Search strategy

A literature review was conducted using PubMed and Google Scholar from June 15th to July 5th, 2025. The search strategy incorporated the following key terms: "bacterial vaginosis", "recurrent bacterial vaginosis", "oral and vaginal probiotics", and "prevention + strategies." 

Studies were eligible if they met the following criteria: focused on non-pregnant women with BV; investigated the use of orally or vaginally administered probiotics as the only or adjunctive treatment; were published in English or Spanish; and were published between 2020 and 2025. Main exclusion criteria included animal studies, studies involving pregnant women, studies involving women with a Virus del Papiloma Humano (VPH) or Human Papillomavirus (HPV) diagnosis, and studies published before 2020. Due to the heterogeneity in study designs and interventions among the included studies and the descriptive nature of this review, a meta-analysis was not performed. All statistical results reported were taken directly from the original studies and referenced meta-analyses, without independent recalculation by the authors.

Etiology, pathophysiology and symptoms

As previously mentioned, BV originates from a dysbiosis in the vaginal bacterial population. A healthy vaginal flora is predominantly composed of bacteria from the genus *Lactobacillus*, with the most common species being *Lactobacillus crispatus, L. gasseri, L. iners, *and *L. jensenii*. These bacteria maintain a healthy environment through various mechanisms, such as keeping the vaginal pH between 3.5 and 4.5. When the population of *Lactobacillus* decreases, the vaginal pH rises, facilitating the proliferation of pathogenic bacteria that are usually present in small quantities [9,10].

These pathogens are mostly anaerobic species such as *Gardnerella vaginalis*, *Prevotella* spp., and *Mobiluncus* spp., many of which exhibit high adherence to the vaginal epithelium. Another important aspect to consider is that *G. vaginalis* also forms biofilms, which are believed to play an important role in the therapeutic failure of BV. These biofilms consist of structured bacterial communities embedded in a self-produced extracellular matrix of polysaccharides and proteins, which also supports the proliferation of other anaerobes, forming communities of different types of bacteria, and protecting them from the immune response and pharmacological agents used in BV treatment [2,8,11]. It also helps bacteria attach to the vaginal epithelial cells [11].

Other anaerobic species implicated in BV pathogenesis also exhibit virulence mechanisms that contribute to persistence and recurrence. For example, *Prevotella* spp. produces proteolytic enzymes and sialidases capable of degrading cervical and vaginal mucins, weakening the mucosal barrier and facilitating pathogen adhesion, nutrient acquisition, and immune evasion. As previously mentioned, these organisms frequently coexist with *G. vaginalis* within complex biofilm structures, where synergistic interactions enhance antimicrobial resistance and promote a dysbiotic environment. [11,12] 

Several risk factors have been associated with the development of BV, including early initiation of sexual activity, having multiple sexual partners, smoking, not using barrier protection such as condoms, usage of intrauterine devices (IUDs), and frequent vaginal douching [1,6].

The most characteristic symptoms include increased vaginal discharge, usually grayish or milky white in color, and a strong odor often described as “fish odor.” In many cases, BV can lead to vaginitis, with symptoms like pruritus, dysuria, and local discomfort. It is important to note that up to 50% of women with BV are asymptomatic [1,3].

Diagnostic criteria

Diagnosis is primarily based on Amsel's criteria, which rely on clinical assessment, and/or the Nugent score, which is derived from Gram staining of a vaginal swab. Gram stain is considered the gold standard for BV diagnosis [1,2].

A diagnosis based on Amsel’s criteria requires at least three of the following four findings: 1) homogeneous, thin, whitish, yellowish, or grayish vaginal discharge; 2) vaginal pH>4.5; 3) presence of clue cells on saline wet mount microscopy; and 4) positive whiff test (fishy odor upon adding 10% potassium hydroxide to the sample) [1,2,13].

The Nugent score evaluates three bacterial morphotypes in the Gram-stained vaginal sample: 1) gram-positive rods (*Lactobacillus*), 2) small gram-negative/variable rods (*G. vaginalis *and *Bacteroides* spp.), and 3) curved gram-variable rods (*Mobiluncus* spp.). Scores range from 0-10: a score of 0-3 indicates normal flora with predominance of lactobacilli, 4-6 indicates intermediate flora with increased *Gardnerella* and *Bacteroides* spp., and 7-10 indicates decreased lactobacilli and abundance of *Gardnerella* and *Bacteroides* spp., and the presence of *Mobiluncus* spp., all of which are compatible with BV [1,9,13].

The diagnosis and treatment of BV are important due to its association with complications. BV increases the risk of acquiring sexually transmitted infections, which may in turn elevate the risk of infertility. It is also linked to pregnancy complications such as premature rupture of membranes and preterm birth [1,2].

Recurrent BV

Recurrent BV is defined as the occurrence of three or more episodes within a 12-month period. Various studies have shown that more than half of women treated for an initial BV episode with antibiotics experience recurrence within six months [8,14].

There are various hypotheses proposed regarding the factors that contribute to the recurrence of BV, such as non-adherence to antibiotic treatment, for example, due to its adverse effects; the sexual transmission of pathogenic bacteria from sexual partners, male or female, known as “reinfection”; or due to vaginal relapse through various mechanisms. Some of the proposed mechanisms for vaginal relapse include the failure of recolonization of the vaginal epithelium with healthy species of *Lactobacillus*, a deficient response from the host immune system, host genetics, and the presence and persistence of the common BV biofilms [5,14,15]. It has been demonstrated that BV biofilms can be present even after completing antibiotic treatment, and the remaining bacteria contained in it can rapidly restore the dysbiotic environment, thus leading to recurrence [11].

Another problem that has been detected is antibiotic resistance. There are studies that have reported various *G. vaginalis* strains that are resistant to both clindamycin and metronidazole, which are the main pillars of BV treatment. This is one of the reasons why it is important to continue evaluating other treatment options beyond the use of antibiotics [15].

Recurrent BV can significantly affect quality of life, specially in patients with multiple recurrences. For instance, a 2023 study assessing quality of life in women with recurrent BV surveyed 62 participants, from different ethnicities, with 61% reporting a negative overall impact, 71% reporting an impact on sexual health, and 75.8% reporting a negative effect on mental health [16]. 

Current treatment options for BV

Currently, the first-line treatment consists of oral or vaginal antibiotics, mainly metronidazole and clindamycin. The Centers for Disease Control and Prevention (CDC) recommends the following regimens: 1) metronidazole 500 mg orally twice daily for seven days, 2) metronidazole gel 0.75%, one full applicator (5 g) intravaginally once daily for five days, or 3) clindamycin cream 2%, one full applicator (5 g) intravaginally at bedtime for seven days. Alternative regimens include clindamycin ovules, oral tinidazole, or oral secnidazole [1,17].

For recurrent BV, extended treatment regimens have been evaluated, such as metronidazole gel 0.75% or 750 mg vaginal suppositories used twice weekly for three months. However, recurrence often occurs upon discontinuation. Other proposed protocols combine various drugs, such as seven days of oral metronidazole followed by intravaginal boric acid plus metronidazole gel given over four to six months [1].

Another important aspect in the treatment of BV is patient education and addressing lifestyle modifications. Current evidence suggests that it is not sufficient to only treat active vaginal infections, but also to promote preventative strategies aimed at maintaining microbial balance. This involves taking into account the hormonal, nutritional, and behavioral factors that influence the composition of the vaginal microbiota [10].

Some of the behavioral factors involve the consistent use of condoms, which prevent the disturbance of normal vaginal microbiota by reducing the entry of exogenous microorganisms and avoiding contact with seminal fluids that can alter vaginal pH [10,15]. Another aspect related to contraception methods and hormonal factors, is hormonal contraceptive use. It has been demonstrated that its use is associated with reduced prevalence, incidence, and recurrence of BV. On the other hand, copper IUDs are associated with an increased risk of BV, so in women who suffer from recurrent BV, it could be beneficial to choose another contraception method [15]. Other behavioral modifications would be the avoidance of vaginal douches, a practice that can still be present among women around the world, and smoking cessation [10,15]. 

Regarding the nutritional aspects, a diet with few simple sugars and a high content of micronutrients, such as vitamins A, C, D, E, folate and calcium, as well as fruits and dairy products, help maintain a healthy vaginal environment [10].

In recent years, attention has shifted toward complementary alternatives, including biofilm-disrupting agents like boric acid, and both oral and vaginal probiotics [1,14,17].

Use of probiotics for BV

Probiotics are defined by the World Health Organization as “live microorganisms which, when administered in adequate amounts, confer a health benefit to the host.” In the context of BV, the probiotics investigated are various species of Lactobacillus [2,13].

One of the primary mechanisms by which lactobacilli help maintain a healthy and protective vaginal environment is through acidification of the vaginal environment via lactic acid production. By maintaining the vaginal pH below 4.5, they inhibit the growth of a wide range of pathogenic bacteria. In addition, lactobacilli produce antimicrobial substances, such as bacteriocins, which act via various mechanisms-including pore formation in bacterial membranes and interference with enzymatic processes essential for cell wall synthesis [2,9,18]. Furthermore, lactobacilli exhibit strong adherence to the vaginal epithelium, creating a physical barrier that protects against pathogens. They also disrupt the *Gardnerella* biofilms by competing for access to nutrients, displacing pathogenic bacteria [9,11,18].

Several recent studies have demonstrated promising results regarding the use of probiotics as an adjunctive therapy for BV, particularly in reducing recurrence rates [2,3,8,13,17]. For example, a randomized, double-blind, placebo-controlled clinical trial evaluated the efficacy of *Lactobacillus crispatus* CTV-05 (Lactin-V) in preventing BV recurrence in 228 women aged 18 to 45 who had previously been treated with 0.75% metronidazole vaginal gel. Participants assigned to the Lactin-V group received the probiotic intravaginally at a dose of 2×10^9^ CFU via prefilled applicators for eleven weeks (once daily for four days during the first week, then twice weekly for the remaining 10 weeks). Recurrence was assessed using Amsel’s criteria and the Nugent score at four, eight, 12, and 24 weeks. At week 12, the recurrence rate was 30% in the Lactin-V group versus 45% in the placebo group. Successful colonization with *L. crispatus* CTV-05 was observed in 79% of treated participants at week 12, with no significant differences in adverse effects between groups. Treatment adherence was also notably high [13].

A systematic meta-analysis including 18 studies and a total of 1,651 patients assessed the efficacy of probiotics in BV treatment. The results showed that the combination of antibiotics and probiotics significantly reduced recurrence rates at both one and three months compared to antibiotics alone. Similarly, probiotics alone, compared to placebo, reduced recurrence and increased cure rates. The analysis also compared treatment durations, concluding that longer probiotic regimens (one to three months) were more effective than shorter ones. Nonetheless, limitations such as small sample sizes and lack of standardization in study design and treatment protocols were emphasized [8]. 

Webb summarized the main probiotic regimens used in recent studies [6]. The Lactobacillus strains evaluated included *L. crispatus; L. acidophilus *GLA-14 + *L. rhamnosus* HN001; *L. fermentum* LF15 + *L. plantarum* LP01, all administered vaginally; and a combination of *L. acidophilus* + *L. rhamnosus* GR-1 + *L. fermentum* RC-14, which was taken orally. Treatment durations ranged from 14 days to six months, either continuously or intermittently. Two of the studies used probiotics as complement to metronidazole therapy, reporting greater reductions in BV recurrence compared to antibiotics alone. The remaining studies evaluated probiotics as monotherapy and also demonstrated reductions in recurrence, although direct comparisons between regimens were limited by differences in study design and population. All the four main studies reviewed showed a reduction in BV recurrence following probiotic treatment [6]. 

A recent meta-analysis by Udjianto et al. evaluated effective probiotic regimens for BV treatment and recurrence prevention [19]. They analyzed 16 randomized controlled trials published between 2014 and 2024. They documented that *L. rhamnosus* TOM 22.8, at a dose of 10 × 10^9^ CFU/day for 10 days, was one of the most effective regimens for improving Nugent scores, lowering vaginal pH, and restoring a vaginal microbiota composed mainly by lactobacilli, making it a very promising strain. Other species and strains that showed favorable results were *L. crispatus* (strains DSM and LMG S-29995), *L. plantarum* (strains 57B, MG989, and PBS067), and *L. acidophilus* (strains W70, KS400, and DDS-1), used either alone or in multi-strain formulations, and their doses varied from 1×10^8^ to 5.4×10^9^ CFU per day. Treatment duration was another of the analyzed variables, and showed that intermediate (one to two months) and long-term (more than two months) treatment duration showed better results. Another aspect that stands out is that the therapeutic efficacy of probiotics for BV was influenced by many factors such as age, disease severity, ethnicity, lifestyle, and geographical differences, which affect baseline microbiota composition. All these factors need to be taken into account when determining the most appropriate regimen for each patient [19]. 

Not only the treatment regimen, but the time of administration is also important, as documented in the study conducted by He et al. [20]. They investigated the effect of *L. rhamnosus* and *L. casei *on the formation of *Gardnerella* spp. biofilms, by administering those species, separately in three different phases of biofilm formation, specifically at 0, 24 and 48 hours, to evaluate their inhibitory effects. They also analyzed the susceptibility of the *Gardnerella* spp. to metronidazole and clindamycin, comparing the planktonic and biofilm-associated bacteria, and the influence of pH on biofilm formation. The results showed that biofilm formation significantly increased resistance to both antibiotics. Most importantly, *L. rhamnosus*, especially when introduced during the early adhesion phase (at zero hours), markedly reduced biofilm thickness and *Gardnerella *load. On the other hand, *L. casei* had a weaker inhibitory effect, especially when added at later stages. Additionally, the biofilm formation was much lower at pH levels below 4.5 or above 6.5, whereas it increased at pH between 4.5 and 6.5. These findings suggest that an early probiotic intervention may be more beneficial than a latter one, especially with *L. rhamnosus*, to disrupt biofilm formation, potentially reduce BV recurrence, and maintain an acidic vaginal environment [20]. 

Another aspect worth highlighting is that most studies reported minimal adverse effects, similar to those observed in placebo groups, supporting the safety of probiotics when used in well-designed regimens [7,8,13]. In other studies, some adverse effects were reported, though they were described as mild or moderate by the participants, and did not lead to any treatment discontinuation. Most common side effects were gastrointestinal symptoms, such as abdominal distension, nausea, bloating, dyspepsia, etc., and were associated with specific strains such as *L. plantarum* P1763. Other side effects that have been reported are vaginal discharge and itching, associated with some *Lactobacillus* spp. products such as inVag and Lactin-V [19]. This is a significant advantage, given that antibiotic-based treatments are often associated with multiple adverse effects, such as nausea, vaginitis, and diarrhea, which can reduce adherence, especially in patients with recurrent BV [6,16]. Moreover, frequent antibiotic use contributes to the growing global problem of antimicrobial resistance [16].

Despite promising findings, other sources note that the current evidence remains insufficient to support a formal recommendation for probiotic use, a position reflected in guidelines such as those from the CDC [1,14]. However, some guidelines, such as those from the Melbourne Sexual Health Centre, acknowledge the promising nature of recent findings, even though conclusive evidence is still lacking [21]. Also, recent review articles, like the one by Joseph et al., have gathered information which support the fact that optimal treatment of BV is not limited to the use of antibiotics alone, but includes comprehensive strategies that restore or maintain a vaginal ecosystem dominated by healthy lactobacilli. However, further clinical research and controlled trials are required to standardize protocols and confirm the safety and efficacy of these interventions [17]. 

Overall quality and limitations of the evidence

The studies included in this review vary considerably in design, sample size, and intervention protocols. While randomized controlled trials such as Cohen et al. [13] provided high-quality evidence supporting the potential efficacy of certain probiotic strains, other trials suffered from methodological limitations, including small sample sizes, short follow-up durations and heterogeneity in probiotic formulations, doses, and administration routes. Outcome definitions were not always standardized, further complicating comparisons across studies. Quality assessments reported in recent meta-analyses such those by Liu et al. [8] and Udjianto et al. [19], have generally rated the evidence as moderate, primarily due to these factors. 

Also, although the available evidence on probiotics for treatment and prevention of recurrent BV is growing, there is a notable lack of studies in resource-limited regions or among diverse demographic groups, which limits the generalizability of the current findings. Future research should also focus on evaluating probiotic interventions in more diverse populations to better understand potential differences in efficacy, accessibility, and implementation across varying healthcare contexts. These limitations should be taken into account when interpreting the current evidence base, and also as an improvement goal for further investigations. 

## Conclusions

BV is a common condition affecting women worldwide, caused by an imbalance in the normal vaginal microbiota. Although antibiotics remain the cornerstone of treatment, they have limited effectiveness in preventing recurrence following treatment completion. Various studies on probiotics, administered alongside or following antibiotic treatment, have yielded promising results in reducing recurrence rates. Nevertheless, current evidence is insufficient to formally recommend probiotics, and no standardized regimen or FDA-approved formulation is yet available. Further well-designed studies with standardized methodologies and long-term follow-up are needed to determine optimal doses, strains, and administration protocols. This would create an opportunity for probiotics to be integrated into international clinical guidelines in the future, while hoping to complement current standard therapies. 
